# Development of an interpretable machine learning model for predicting venous thromboembolism in intensive care unit patients with intracerebral hemorrhage

**DOI:** 10.3389/fneur.2025.1691549

**Published:** 2026-01-07

**Authors:** Menghui He, Wenyan Liu, Zhongsheng Lu, Yiwei Lv, Qiang Zhang, Xiaoqing Jin, Pei Han

**Affiliations:** 1 Department of Graduate School, Qinghai University, Xining, China; 2 Department of Neurosurgery, Qinghai Provincial People’s Hospital, Xining, China

**Keywords:** intracerebral hemorrhage, machine learning, prediction model, SHAP, venous thromboembolism, XGBoost

## Abstract

**Background:**

Venous thromboembolism (VTE) is a frequent and potentially life-threatening complication in patients with intracerebral hemorrhage (ICH) in intensive care units (ICU). However, the necessity of prophylactic anticoagulation therapy for these patients remains controversial. This study aims to develop an interpretable machine learning (ML) model to accurately predict the risk of VTE in critically ill ICH patients, thereby enabling timely and individualized preventive measures.

**Methods:**

A retrospective analysis was performed on clinical data from the MIMIC-IV database and ICU patients diagnosed with ICH at Qinghai Provincial People’s Hospital. After data preprocessing, 1,545 cases from the MIMIC-IV database were randomly divided into a training set (1,097 cases) and a test set (448 cases) in a 7:3 ratio. Data from 151 ICH patients treated in the ICU of Qinghai Provincial People’s Hospital between January 2020 and December 2024 were utilized as an external validation set. The Least Absolute Shrinkage and Selection Operator (LASSO) algorithm was applied for feature selection. Model performance was assessed using metrics including the area under the curve (AUC), decision curve analysis (DCA), accuracy, positive predictive value (PPV), and negative predictive value (NPV). The optimal model was further explained using the SHapley Additive exPlanations (SHAP) method.

**Results:**

The XGBoost model exhibited the best predictive performance, with AUC values of 0.936, 0.778, and 0.761 for the training set, test set, and external validation set, respectively. Feature importance analysis identified the top 10 influential features as follows: ICU stay duration, age, prothrombin time, triglycerides, albumin, body mass index, partial thromboplastin time, blood glucose, white blood cell count, and systolic blood pressure.

**Conclusion:**

The XGBoost model accurately predicts VTE occurrence in ICH patients in the ICU. By employing the SHAP method, it is possible to precisely assess the impact of various pathophysiological parameters on individual patient predictions, thereby providing robust support for personalized risk stratification and preventive treatment.

## 1 Introduction

Patients with intracerebral hemorrhage (ICH) are frequently at risk of venous thromboembolism (VTE)—a severe complication that primarily presents as deep vein thrombosis (DVT) or pulmonary embolism (PE) (1). Studies indicate that approximately 3% of ICH patients develop DVT or PE, with the incidence of symptomatic VTE ranging between 1 and 10% (2). Beyond prolonging hospital stays and increasing healthcare costs, VTE also significantly elevates mortality risk (3). Prophylactic anticoagulation is an established effective strategy to reduce VTE incidence (4). Despite the critical need for VTE prevention in ICH patients, a significant clinical conflict exists between anticoagulation and hemostatic therapies. Clinicians must consider not only the risk of fatal VTE in the absence of anticoagulation but also the risk of intracranial hematoma expansion potentially induced by anticoagulation (5). Balancing these dual therapeutic priorities and developing a scientifically sound intervention strategy for this patient population has emerged as a critical, unmet challenge in clinical practice.

Precise, adaptable assessment tools are critical for the early identification of severe intracerebral hemorrhage (ICH) patients at risk of venous thromboembolism (VTE). Relative to traditional approaches, machine learning (ML) algorithms can discern latent associations and patterns within complex medical datasets. Through the learning and analysis of large-scale, complex datasets, such algorithms contribute substantially to clinical disease diagnosis and outcome assessment (6). In recent years, the application of ML in clinical medicine has grown increasingly prevalent; specifically, ML is employed to quantify risks, identify predictive factors, and develop high-precision diagnostic and prognostic models (7).

This study employed five machine learning models, including XGBoost, Logistic Regression (LR), Random Forest (RF), K-Nearest Neighbors (KNN), and Support Vector Machine (SVM), to construct a predictive framework for the risk of VTE in severe ICH patients. The research aims to thoroughly explore the risk factors for VTE occurrence through interpretable machine learning methods, providing a scientific basis for clinical decision-making.

## 2 Materials and methods

### 2.1 Data source

The data for this study were sourced from the Medical Information Mart for Intensive Care IV (MIMIC-IV) (Record ID: 66983242) and from patients with ICH admitted to the ICU of Qinghai Provincial People’s Hospital. Specifically, the data extracted from the MIMIC-IV database were randomly divided into a training set (70%) and a test set (30%). The data collected from the ICU of Qinghai Provincial People’s Hospital were used as an external validation set.

MIMIC-IV is a publicly accessible intensive care database that comprises clinical data from inpatients at Beth Israel Deaconess Medical Center (BIDMC) between 2008 and 2019 (8). This database systematically documents patients’ vital signs, surgical procedures, laboratory test results, comorbidities, medication records, demographic information, and follow-up survival status. Given that patient identifiers have been de-identified in the database, informed consent was not required for its use. Additionally, we collected data from patients with ICH admitted to the ICU of Qinghai Provincial People’s Hospital between January 2020 and December 2024. This study was approved by the Research Ethics Committee of Qinghai Provincial People’s Hospital (reference number: 2025–400-01). All participants or their legal guardians were informed and provided consent to participate in this study. Throughout the research process, all methods and procedures were conducted strictly with the Declaration of Helsinki principles. Patient data were anonymized, with no patient-identifiable information recorded.

### 2.2 Study population and target variables

**Figure 1:**
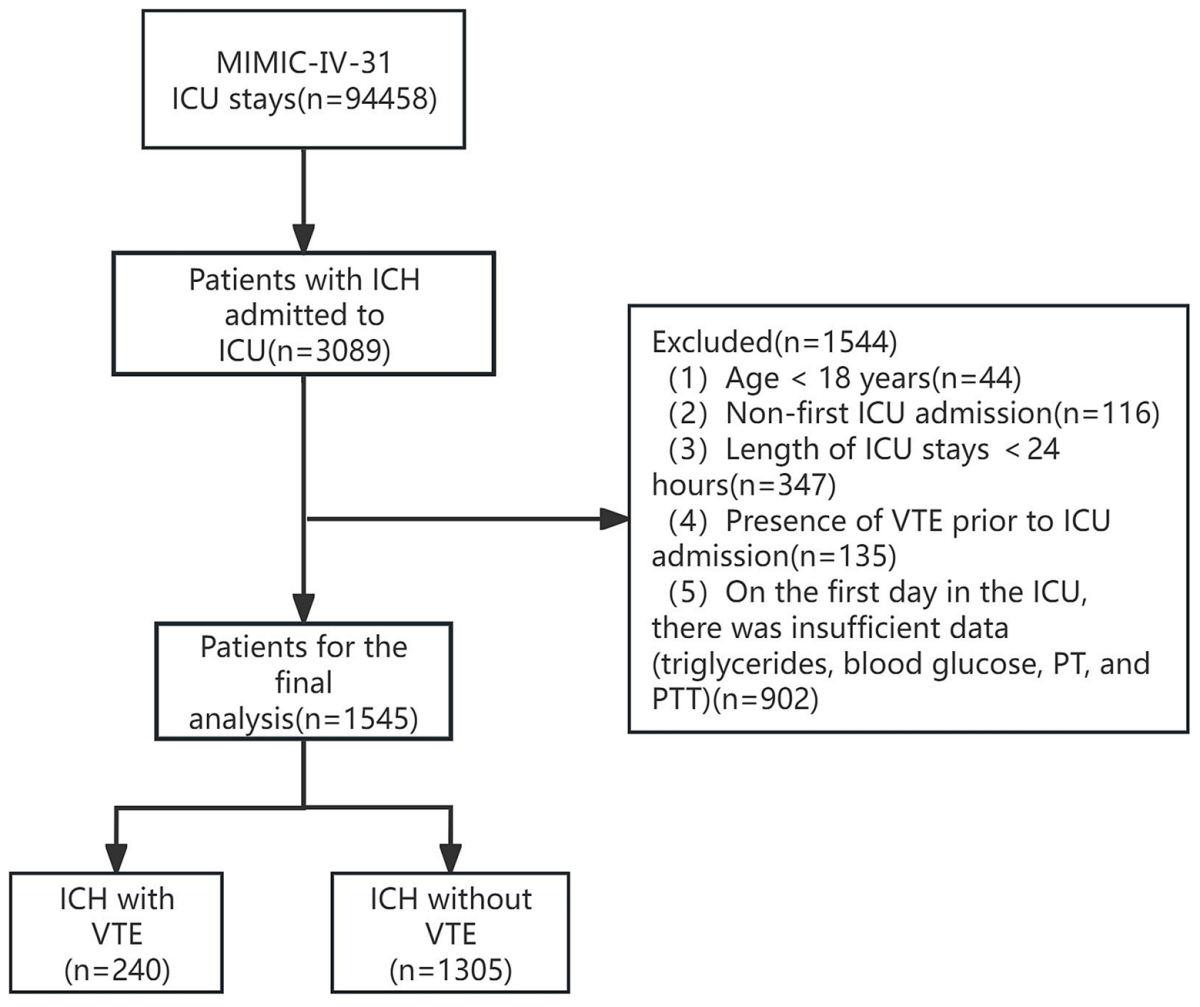
The flow chart of patient selection. The study enrolled 1,545 patients with severe ICH.

Patients with ICH admitted to the ICU were included in the study cohort. Samples with missing variables exceeding 20% were excluded. The retrieval of ICH patients utilized the diagnostic codes from the International Classification of Diseases, Ninth Revision (ICD-9) and Tenth Revision (ICD-10). The screening process for patient inclusion in this study is illustrated in Figure 1. Exclusion criteria included: (1) age < 18 years; (2) patients not admitted to the ICU for the first time; (3) ICU stay duration less than 24 h; (4) presence of venous thromboembolism at admission; (5) insufficient data on the first day of admission (including triglycerides, blood glucose, prothrombin time, and partial thromboplastin time).

VTE is defined as DVT (including upper or lower extremities) and/or PE. This study extracted VTE data based on ICD-9 and ICD-10 codes from the MIMIC-IV database. Additionally, we manually reviewed bilateral limb venous ultrasound, CT venography, or CT pulmonary angiography (CTPA) reports of ICH patients in the intensive care unit of Qinghai Provincial People’s Hospital to confirm the diagnosis of VTE. Imaging examinations are performed as part of the routine screening protocol, and the date of VTE diagnosis is determined by the date of imaging confirmation.

### 2.3 Data extraction and preprocessing

41 predictive features were extracted through Structured Query Language (SQL). All laboratory data were collected at the time of hospital admission. Variable collection can be categorized into four groups: (1) Baseline characteristics: gender, age, BMI (weight/height^2^), ICU stay duration; (2) Vital signs: heart rate, systolic blood pressure (SBP), diastolic blood pressure (DBP), respiratory rate, body temperature, oxygen saturation (Spo2), Glasgow Coma Scale (GCS), SOFA score; (3) Biochemical indicators (collected within 24 h of admission): blood urea nitrogen (BUN), serum potassium, serum sodium, blood glucose, creatinine level, white blood cell count (WBC), red cell distribution width (RDW), red blood cell count (RBC), platelet count, mean corpuscular volume (MCV), hemoglobin, hematocrit, mean corpuscular hemoglobin (MCH), mean corpuscular hemoglobin concentration (MCHC), international normalized ratio (INR), prothrombin time (PT), partial thromboplastin time (PTT), albumin, serum calcium, triglycerides; (4) Complications: VTE (deep vein thrombosis and pulmonary embolism), congestive heart failure (CHF), chronic obstructive pulmonary disease (COPD), renal disease, liver disease, atrial fibrillation, peripheral vascular disease, paraplegia, malignant cancer.

To address the issue of missing data, this study employed Multivariate Imputation techniques for data filling. Specifically, we employed the Multiple Imputation by Chained Equations (MICE) algorithm to achieve this purpose. During the variable preprocessing stage, continuous data were transformed using standardization methods, while categorical variables were factorized. To address the issue of class imbalance, this study employed the Random Over-Sampling Examples (ROSE) method. We implemented this algorithm using the ROSE package in R language, adhering to the package’s default settings where the appropriate sampling ratio is automatically determined based on the degree of dataset imbalance. This approach helps enhance the model’s generalization capability, enabling accurate predictions for both majority and minority classes.

### 2.4 Construction of the machine learning model

This study constructed five prediction models using the training dataset: XGBoost (implemented via the xgboost R package v1.7.5.1 in R 4.4.2, using tree-based booster [gbtree] for binary classification), logistic regression (LR), random forest (RF; with fixed random seed of 12,345 and five-fold cross-validation partitioning before bagging), K-nearest neighbors (KNN), and support vector machine (SVM). Model training and hyperparameter optimization were performed through five-fold repeated cross-validation. In this process, XGBoost employs grid search to optimize key parameters and implements an early stopping mechanism (training stops if the validation set AUC improvement is less than 0.001 for 10 consecutive iterations) to prevent overfitting. The test set and external validation set remain completely isolated during the training process, serving solely for final model validation after determination. This approach effectively reduces overfitting risks and ensures independence in model selection and evaluation.

### 2.5 Model assessment

Based on the area under the receiver operating characteristic curve (AUC), this study systematically evaluated the predictive performance of five machine learning algorithms in the test set. By comprehensively examining the performance parameters of each model and integrating the results of decision curve analysis (DCA), the machine learning algorithm with the best overall performance was ultimately selected. Subsequently, the calibration performance of this model was validated, and patient data from Qinghai Provincial People’s Hospital were used as an external validation group to further assess the model’s stability and generalization capabilities.

### 2.6 Machine learning explainable tool

We employed the SHAP method to interpret the predictive model, which precisely quantifies the contribution and influence of each feature on the final prediction outcome. Notably, SHAP not only accounts for the independent contribution of features but also assesses interactions among these features, thereby providing a more comprehensive explanation. To assess feature SHAP values, we utilized SHAP feature importance plots, SHAP scatter plots, and SHAP waterfall plots. These visualization approaches help delineate the predictive mechanisms of the “black-box” model, thereby enhancing the model’s transparency and credibility.

### 2.7 Statistical analysis

The statistical processing and data visualization in this study were conducted using R language (version 4.4.2). Differentiated approaches were adopted based on the characteristics of the variables when selecting the analytical methods. For categorical data, chi-square tests or Fisher’s exact tests were employed for statistical analysis, with results presented as a combination of frequencies and percentages. Different strategies were applied to continuous variables according to their distribution characteristics. Data conforming to a normal distribution were described using means combined with standard deviations, and group comparisons were performed using t-tests. For data not conforming to a normal distribution, quartiles were used for description, and the Wilcoxon rank-sum test was applied for difference testing. Regarding the significance level, *p* < 0.05 was set as the criterion for statistical significance.

This study employs LASSO regression analysis to standardize the statistical analysis results and eliminate potential confounding factors. This method enhances the predictive accuracy and interpretability of statistical models, particularly suitable for dimensionality reduction in high-dimensional data. To ensure minimal autocorrelation, we selected features with non-zero coefficients from the LASSO regression model for additional analysis. The discriminative performance of the model is quantified by AUC, supplemented by metrics such as sensitivity and accuracy for comprehensive evaluation. To further validate the clinical applicability of the model, DCA is used to calculate the net benefit values at different risk thresholds, assessing the decision utility of the predictive model.

## 3 Results

### 3.1 Patient characteristics

This study extracted 3,089 cases of ICH from the MIMIC-IV database. After applying the exclusion criteria, 1,545 patients were ultimately included. The data from the MIMIC-IV database were randomly divided into a training set (*n* = 1,097) and a test set (*n* = 448) in a 7:3 ratio, which were used for model development and performance evaluation, respectively. The data from Qinghai Provincial People’s Hospital served as the external validation set (*n* = 151). Prior to applying the ROSE algorithm, the training dataset exhibited significant class imbalance, with 172 venous thromboembolism (VTE) cases (minority class) and 925 non-VTE cases (majority class). After ROSE-based balancing treatment, the training set achieved a nearly balanced distribution (545 VTE cases vs. 552 non-VTE cases), effectively alleviating the issue of insufficient minority class samples. The comparison of baseline characteristics between the training set and the test set showed no statistically significant differences in any of the indicators (Supplementary Table 1). Further analysis of intergroup differences in the training set regarding the occurrence of VTE (Table 1) revealed significant disparities in age (*p* < 0.001), presence of malignancy (*p* = 0.008), length of ICU stay (*p* < 0.001), and several biochemical indicators (such as hemoglobin, prothrombin time, albumin, and red cell distribution width, etc.).

**Table 1:** Characteristics of ICH patients in the training set.

Characteristic	ICH without VTE	ICH with VTE	*p*-value
*N*	925	172	
Gender			0.803
Female	415 (44.9%)	79 (45.9%)	
Male	510 (55.1%)	93 (54.1%)	
Atrial fibrillation			0.784
No	660 (71.4%)	121 (70.3%)	
Yes	265 (28.6%)	51 (29.7%)	
CHF			0.826
No	769 (83.1%)	142 (82.6%)	
Yes	156 (16.9%)	30 (17.4%)	
Liver_disease			0.667
No	842 (91.0%)	155 (90.1%)	
Yes	83 (9.0%)	17 (9.9%)	
Peripheral vascular disease			0.210
No	859 (92.9%)	155 (90.1%)	
Yes	66 (7.1%)	17 (9.9%)	
COPD			0.182
No	805 (87.0%)	143 (83.1%)	
Yes	120 (13.0%)	29 (16.9%)	
Paraplegia			0.671
No	566 (61.2%)	102 (59.3%)	
Yes	359 (38.8%)	70 (40.7%)	
Renal disease			0.242
No	782 (84.5%)	152 (88.4%)	
Yes	143 (15.5%)	20 (11.6%)	
Malignant cancer			0.008
No	843 (91.1%)	145 (84.3%)	
Yes	82 (8.9%)	27 (15.7%)	
Age	55.01 ± 18.13	60.54 ± 15.39	<0.001
ICU stay	5.69 ± 6.05	10.44 ± 9.70	<0.001
BMI	28.56 ± 7.25	28.80 ± 7.56	0.472
Heart rate	84.24 ± 17.88	86.91 ± 20.51	0.119
SBP	133.74 ± 24.91	130.67 ± 24.11	0.123
DBP	77.15 ± 20.22	72.86 ± 16.21	0.004
Respiratory rate	19.14 ± 5.20	19.65 ± 5.61	0.204
Body temperature	36.80 ± 0.78	36.89 ± 0.69	0.105
Spo2	97.16 ± 3.65	97.43 ± 3.06	0.346
BUN	21.41 ± 15.55	21.30 ± 14.87	0.978
Serum potassium	4.03 ± 0.68	4.11 ± 0.66	0.087
Serum sodium	138.95 ± 5.03	139.57 ± 4.79	0.170
Blood glucose	147.84 ± 65.66	141.86 ± 55.67	0.993
Cr	1.18 ± 0.94	1.08 ± 0.72	0.611
WBC	11.17 ± 5.54	11.46 ± 6.10	0.773
RDW	14.37 ± 1.93	14.85 ± 2.20	0.004
RBC	4.02 ± 0.79	3.82 ± 0.79	0.005
Platelet count	210.03 ± 90.79	208.14 ± 97.10	0.483
MCV	91.30 ± 7.17	90.47 ± 7.60	0.233
Hemoglobin	12.07 ± 2.27	11.24 ± 2.32	<0.001
Hematocrit	36.43 ± 6.46	34.29 ± 6.57	<0.001
MCH	30.19 ± 2.69	29.54 ± 2.72	0.002
MCHC	33.09 ± 1.61	32.70 ± 1.54	0.002
INR	1.28 ± 0.48	1.50 ± 1.09	<0.001
PT	14.01 ± 4.57	15.59 ± 5.43	<0.001
PTT	30.55 ± 9.88	31.82 ± 9.56	0.062
Albumin	3.42 ± 0.66	3.08 ± 0.81	<0.001
Serum calcium	8.62 ± 1.06	8.65 ± 0.91	0.326
Triglycerides	163.23 ± 107.41	152.50 ± 72.83	0.633
GCS	13.95 ± 2.11	14.02 ± 2.17	0.175
SOFA	1.34 ± 1.93	1.26 ± 1.77	0.573

CHF, congestive heart failure; COPD, chronic obstructive pulmonary disease; BMI, body mass index; SBP, systolic blood pressure; DBP, diastolic blood pressure; Spo2, oxygen saturation; BUN, blood urea nitrogen; Cr, creatinine; WBC, white blood cell count; RDW, red cell distribution width; RBC, red blood cell count; MCV, mean corpuscular volume; PT, prothrombin time; INR, international normalized ratio; PTT, partial thromboplastin time; GCS, Glasgow Coma Scale.

### 3.2 Selection of predictors

**Figure 2:**
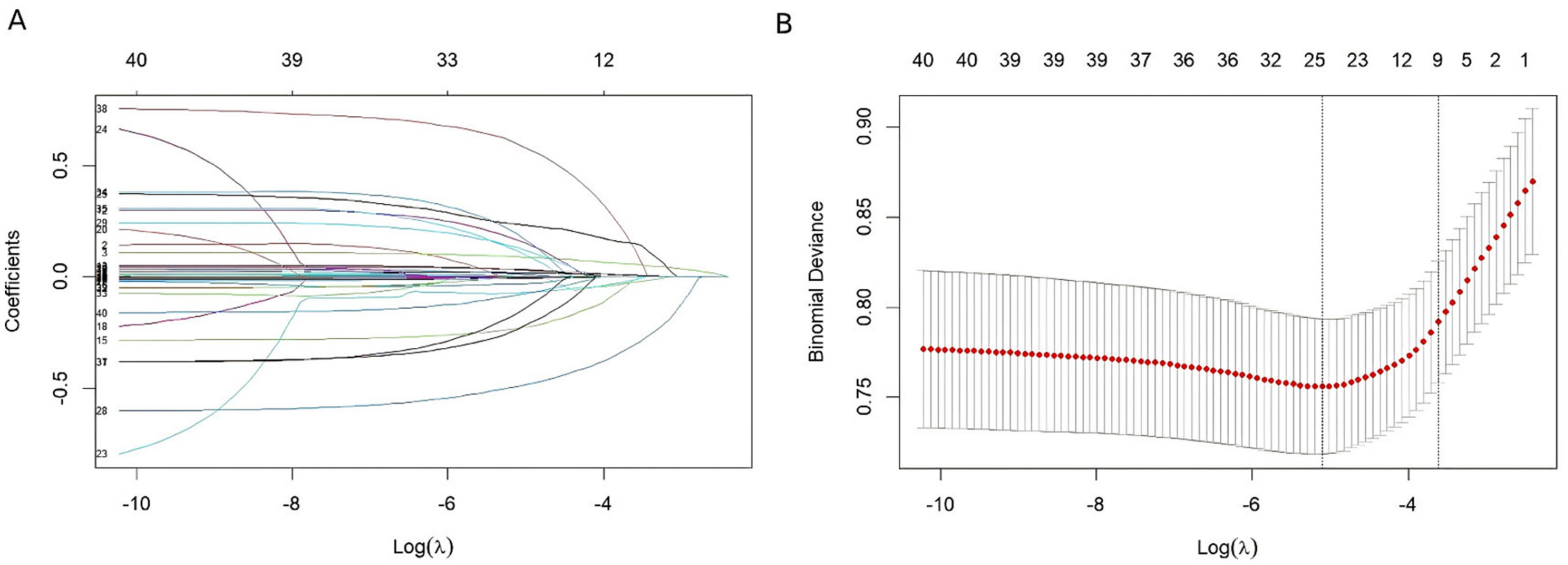
The result of the Least Absolute Shrinkage and Selection Operator (LASSO) method for filtering variables. **(A)** Coefficients of all predictors gradually returning to zeros by used 10-fold cross-validation. **(B)** 10 predictors with non-zero coefficients at the rightmost dashed line.

In the LASSO method, the regularization degree of the *β* coefficients is determined by the tuning parameter *λ* (λ = 0.02690252; lambda.1se; Figure 2). Through feature screening, 10 key variables with non-zero coefficients were ultimately identified, including ICU stay duration, age, prothrombin time, triglycerides, albumin, body mass index, partial thromboplastin time, blood glucose, white blood cell count, and systolic blood pressure.

### 3.3 Model performance

**Figure 3:**
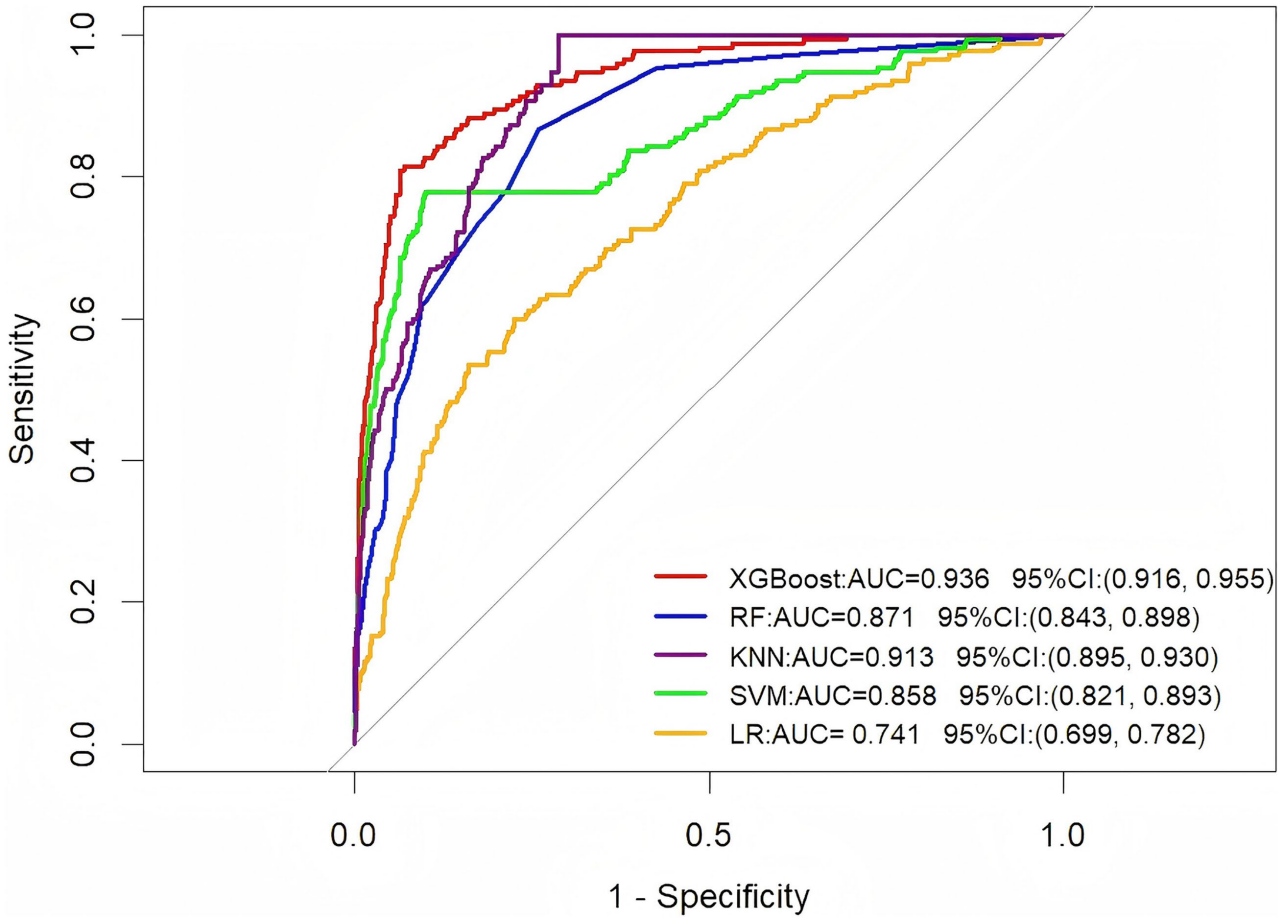
ROC curve analysis of five machine learning algorithms in the training set for predicting VTE in ICH patients.

**Figure 4:**
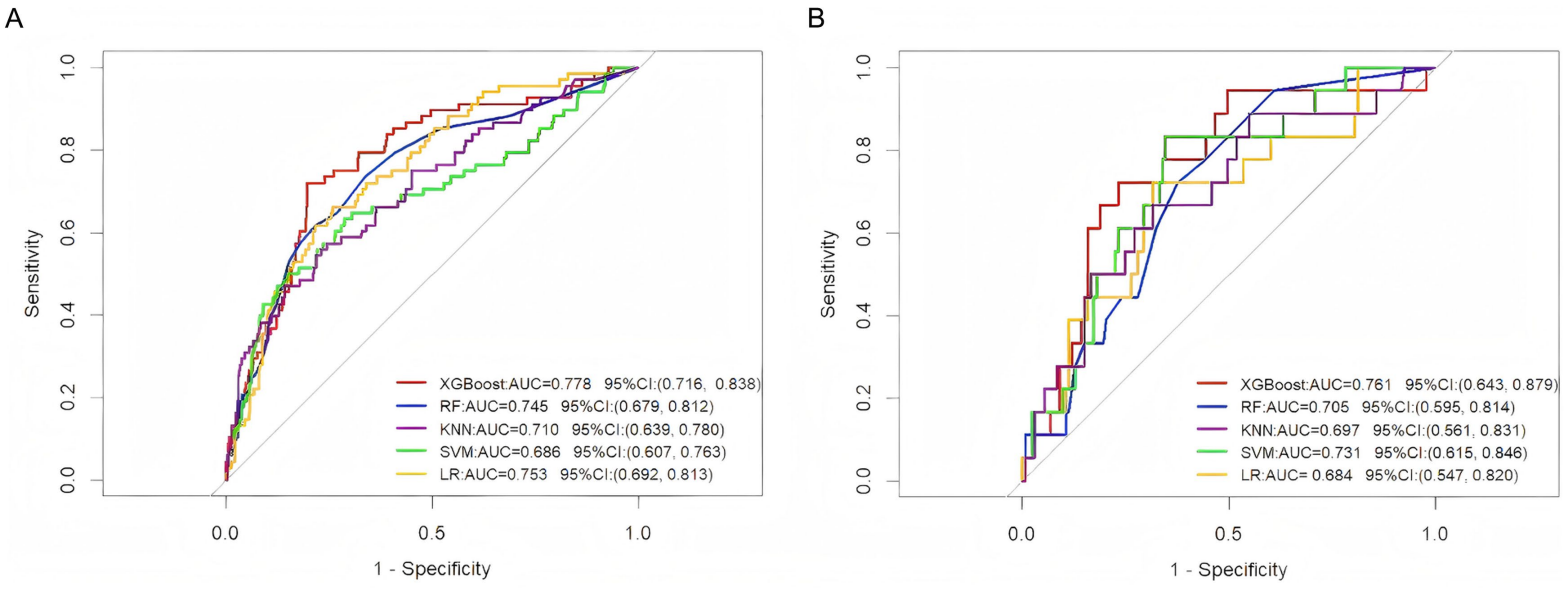
**(A)** ROC curve analysis of five machine learning algorithms in the test set for predicting VTE in ICH patients. **(B)** ROC curve analysis of five machine learning algorithms in the external validation set for predicting VTE in ICH patients.

This study constructed five predictive models, namely XGBoost, LR, RF, KNN, and SVM, based on the training set (Figure 3). In the test set, the XGBoost model demonstrated the best predictive performance, with an AUC value of 0.778 and a 95% confidence interval of (0.716, 0.838). In contrast, the SVM model showed relatively weaker predictive capability, with an AUC value of 0.686 and a 95% confidence interval of (0.607, 0.763). The ROC curves and AUC values of different models in the test set and external validation set are shown in Figures 4A,B. To comprehensively evaluate the model performance, this study employed multiple evaluation metrics, including accuracy, sensitivity, positive predictive value (PPV), negative predictive value (NPV), and F1 score (Table 2). On the external validation set, the AUC value of XGBoost remained stable at 0.761 (95% CI 0.643, 0.879). Although its confidence interval slightly widened compared to the training set, it was still significantly higher than the random prediction baseline (AUC = 0.5). These results indicate that the predictive model demonstrates stable generalization performance, and its output results are in good agreement with real clinical scenarios, providing practical technical support for quantitative disease risk assessment.

**Table 2:** Predictive performance of the models.

Model	AUC (%)	Sensitivity (%)	F1score	Accuracy (%)	PPV	NPV
XGBoost	0.778	0.647	0.717	0.864	0.798	0.842
RF	0.745	0.426	0.545	0.732	0.764	0.682
LR	0.753	0.486	0.504	0.729	0.523	0.758
SVM	0.686	0.365	0.493	0.784	0.782	0.741
KNN	0.710	0.424	0.548	0.734	0.770	0.752

AUC, area under the curve; PPV, positive predictive value; NPV, negative predictive value; XGBoost, extreme gradient boosting; RF, random forest; LR, logistic regression; SVM, support vector machine; KNN, K-nearest neighbors.

**Figure 5:**
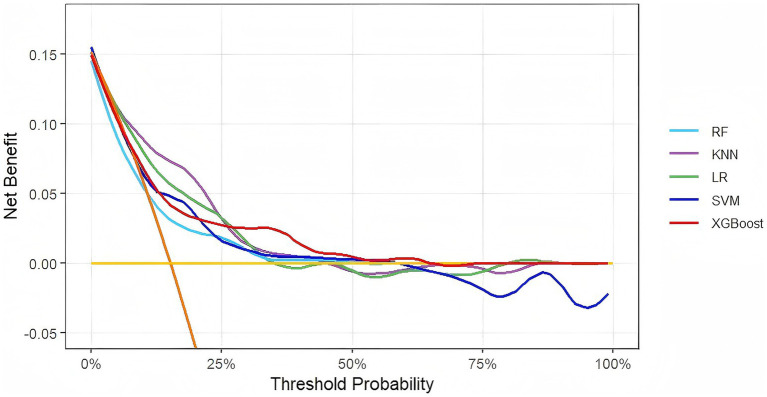
Decision curve analysis of five models plotting net benefits with different threshold probabilities.

**Figure 6:**
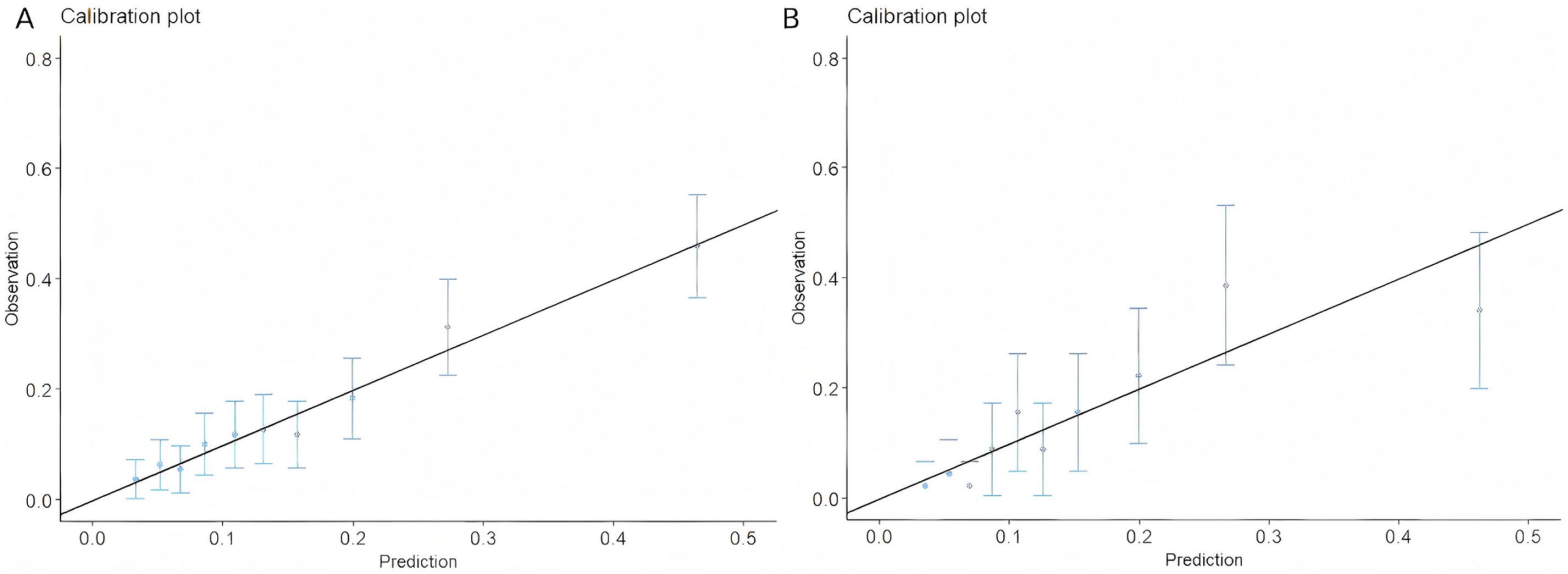
Calibration plots of the XGBoost model in the training **(A)** and testing sets **(B)**.

DCA was used to evaluate the clinical utility of each model, with a focus on net clinical benefit performance across different probability thresholds (Figure 5). Analytical data indicated that all five models exhibited superior clinical decision-making value compared to the two extreme strategies: “treating all patients” (orange reference line) and “treating no patients” (yellow reference line). Notably, the XGBoost model sustained the highest net benefit across most probability threshold ranges, demonstrating exceptional clinical application potential. From the calibration curve (Figure 6), it can be observed that the predicted outcomes of the XGBoost model on both the training set and test set were highly consistent with actual outcomes. Based on multi-dimensional evaluation results, XGBoost was ultimately selected as the core model for predicting VTE in patients with ICH.

### 3.4 Interpretation of personalized predictions

**Figure 7:**
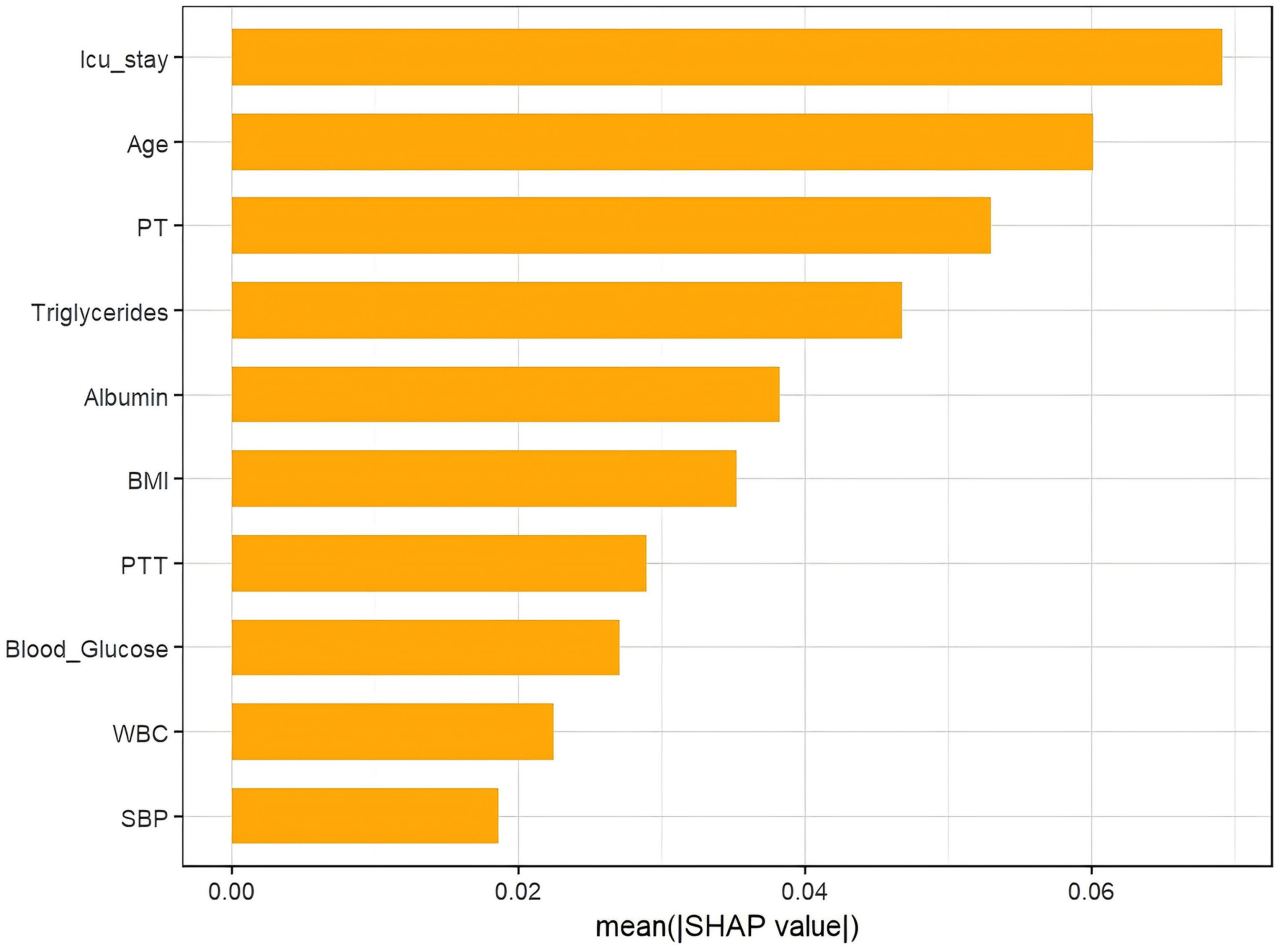
The weights of variables importance.

**Figure 8:**
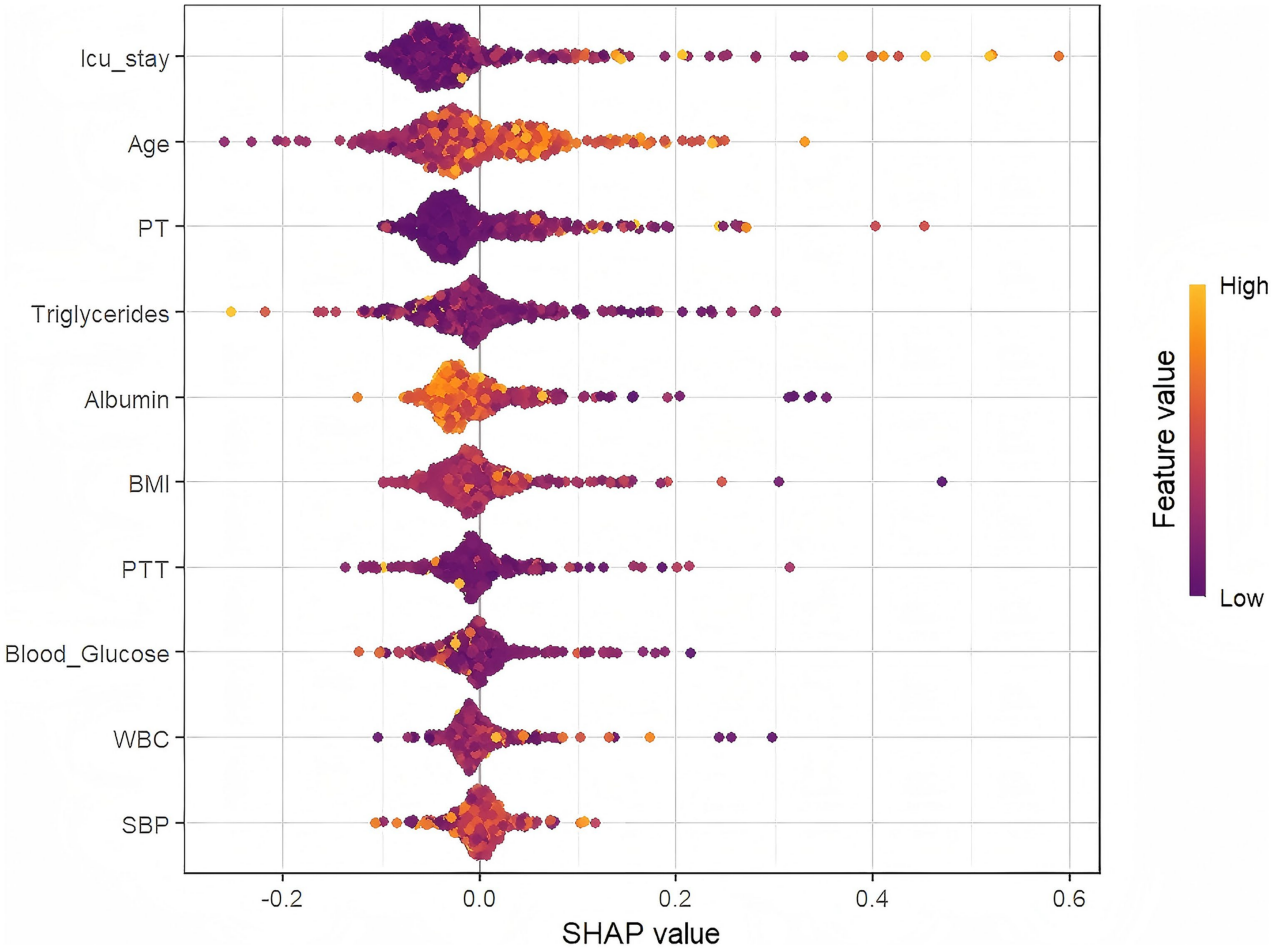
Scatter plot of feature values and SHAP values.

As shown in Figure 7, ICU stay duration is the most influential predictor of VTE occurrence, followed by Age, PT, Triglycerides, Albumin, BMI, PTT, Blood Glucose, WBC, and SBP. Figure 8 further elucidates the directional impact of each variable. For instance, Age exhibits a strong positive correlation with adverse outcomes, with higher values (right side, orange) associated with increased risk. Conversely, higher Albumin levels (left side, orange) are linked to reduced risk, as reflected by the negative SHAP values.

**Figure 9:**
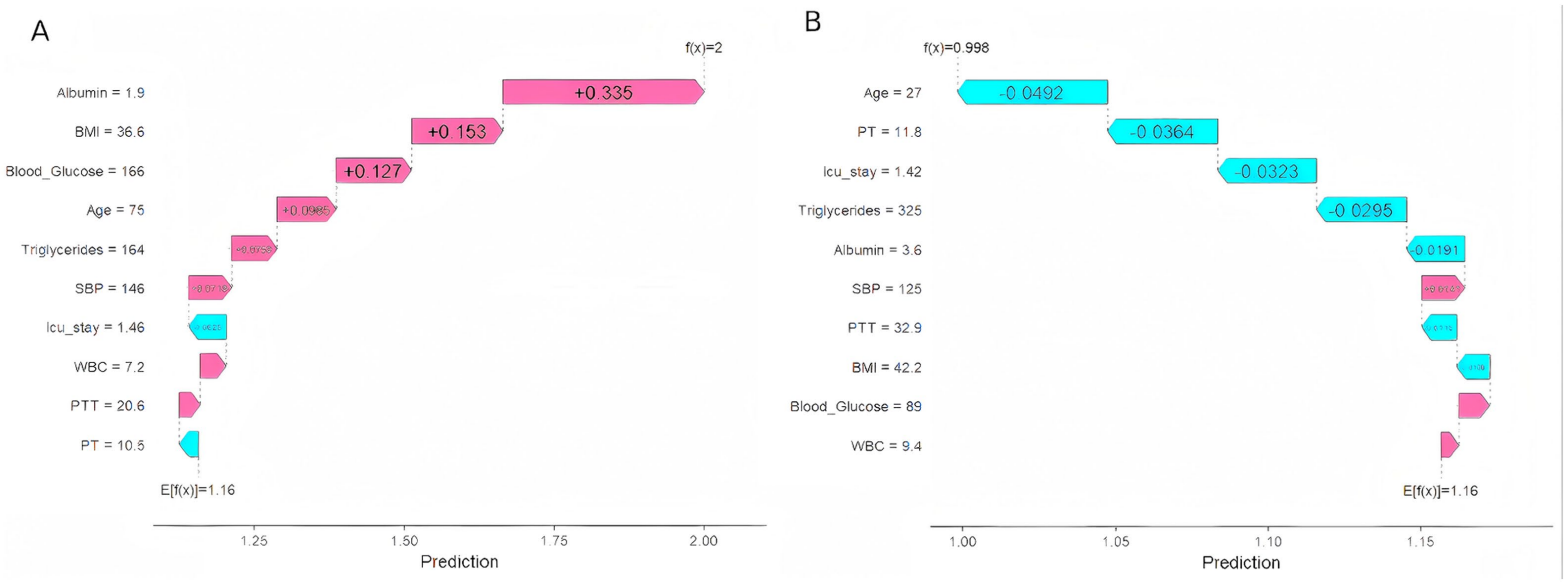
SHAP waterfall plots for two selected patients: **(A)** Patient with VTE occurrence. **(B)** Patient without VTE occurrence.

Figure 9 displays individual SHAP waterfall plots for two patients: one who developed VTE (Figure 9A) and one who did not (Figure 9B). In the figure, red arrows indicate features that increase risk, while blue arrows denote features that suppress risk. The length of the arrows corresponds to the magnitude of the feature contributions. For Patient A (who developed VTE), high-risk features such as hypoalbuminemia (Albumin = 1.9), extreme obesity (BMI = 36.6), and advanced age (Age = 75) collectively elevated the predicted value [f(x) = 2.0] above the baseline E[f(x)] = 1.16, strongly indicating a poor outcome. Conversely, for patient B (who did not develop VTE), the protective characteristics predominated, pushing the predicted value [f(x) = 0.998] below the baseline E[f(x)] = 1.16.

## 4 Discussion

Globally, the incidence of ICH is approximately 29.9 per 100,000 person-years, and this rate has not decreased significantly over the past four decades (9). Following ICH onset, the body’s coagulation cascade is activated, triggering local platelet aggregation and subsequent microthrombus formation. This process not only impairs cerebral hematoma absorption but also may induce adverse events such as VTE (10). Thus, timely identification of VTE risk factors in ICH patients and targeted interventions for effective prevention and management are critical for improving patient prognosis. Currently, commonly used thromboembolism risk assessment tools, such as the Padua Prediction Score, Improved Risk Score for VTE in Stroke, Caprini Score, and Wells Score, exhibit significant limitations when applied to ICH patients. Most of these tools were developed for mixed stroke populations (ischemic and hemorrhagic) or surgical patients and have not been adequately validated in cohorts of pure ICH (11). Traditional approaches such as univariate and multivariate logistic regression have limited accuracy in outcome prediction. For example, Ma et al. (12)developed a traditional binary logistic regression model to predict VTE in ICH patients, using spontaneous echo contrast (SEC), albumin, and age as predictors. While the model performed well, the authors noted a key limitation: the exclusion of artificial intelligence (AI) integration. In recent years, ML algorithms have been widely applied in medical research and frequently outperform traditional statistical models.

This study systematically evaluated the performance of multiple machine learning algorithms and, for the first time, validated the superiority of the XGBoost model in predicting VTE events in patients with severe ICH (test set AUC = 0.778; external validation set AUC = 0.761). Experimental results confirmed that the model’s strong performance originated from inherent predictive validity rather than overfitting. Through machine learning methods, the researchers achieved precise prediction of VTE risk in ICH patients, providing a scientific basis for clinical formulation of individualized prophylactic anticoagulation strategies, which helps optimize patient prognosis and improve their quality of life. Using SHAP waterfall plot analysis, the study revealed the contribution of core pathophysiological indicators to individual prediction outcomes, enhancing the model’s interpretability. Through a systematic evaluation based on the SHAP method, the research team not only quantified the clinical weights of predictive variables but also conducted an integrated analysis based on the importance ranking and clinical characteristics.

Our research findings indicate that in the model interpretability analysis, ICU length of stay, age, and BMI demonstrated high SHAP values. This suggests that these factors play a crucial role in predicting the risk of VTE in patients with severe ICH. This finding is consistent with previous research results. It is noteworthy that the length of ICU stay may have a bidirectional association with venous thromboembolism (VTE): on one hand, prolonged hospitalization increases VTE risk, which is related to immobilization, systemic inflammatory response, and invasive interventions, and is also consistent with its role as the primary predictive factor in the model; on the other hand, the occurrence of VTE may prolong ICU stay due to additional anticoagulation therapy and complication monitoring. The study by Quanhong Chu et al. (13) showed that age >60 years (OR: 2.138, 95% CI: 1.087–4.207, *p* = 0.028) and hospital stay duration >16 days (OR: 2.548, 95% CI: 1.381–4.701, *p* = 0.003) are independent risk factors for VTE in ICH patients. Moreover, our research indicates that BMI is also an independent risk factor for VTE. Existing studies have shown that the risk of VTE in obese patients (BMI ≥ 30 kg/m^2^) is 1.5–2.7 times higher than that in individuals with normal weight (HR/RR: 1.62–2.74) (14). The study by Kim et al. (15) further quantified the relationship between BMI and VTE risk, demonstrating that for every 5 kg/m^2^ increase in BMI, the risk of VTE increases by 50%. Prolonged hospitalization, advanced age, and obesity all significantly increase the risk of VTE. In response to these high-risk factors, clinicians should adopt comprehensive measures, including early mobilization, mechanical prophylaxis, pharmacological prophylaxis, and lifestyle interventions, to reduce the incidence of VTE and improve patient outcomes.

It is noteworthy that abnormalities in coagulation function indicators are also closely associated with the occurrence of VTE in ICH patients. PT and APTT are key indicators for assessing the extrinsic and intrinsic coagulation pathways, respectively. The relationship between PT and the risk of VTE in ICH patients is complex and highly context-dependent, far from being a simple positive or negative correlation. One study has shown that a shortened PT is associated with the occurrence of VTE (16). In contrast, multiple studies on hospitalized COVID-19 patients consistently found that prolonged PT (e.g., exceeding baseline by 3 s) is an independent predictor of VTE (17, 18). The contradictory nature of these results may stem from the heterogeneity of the study populations (e.g., whether they had COVID-19) and the interference of potential confounding factors. Although theoretically a shortened PT may indicate a hypercoagulable state, the clinical evidence supporting it as an independent risk factor for VTE is weak and fraught with contradictions. Its predictive power and strength of evidence are far inferior to those of a shortened APTT. Multiple studies have shown that a shortened APTT is a marker of a hypercoagulable state, potentially caused by elevated levels or activation of coagulation factors (such as VIII, XI, etc.) (19, 20). APTT is currently more widely used in clinical practice and, compared to PTT, it includes specific activators that allow for a more precise measurement of the activity of certain coagulation components. Since the MIMIC-IV database does not provide data on APTT, we only analyzed PTT and found that a shortened PTT is independently associated with secondary VTE in ICH patients, which is also a limitation of our study design.

Studies have shown that the risk of VTE in patients with ICH complicated by hypertension is significantly increased (5, 21). This association may be related to vascular endothelial dysfunction, enhanced inflammatory response, and hypercoagulable state induced by hypertension (22, 23). Additionally, patients with hypertension often have other metabolic diseases (such as diabetes and hyperlipidemia), which further increase the risk of thrombosis (24). There are few studies on the impact of blood glucose levels on VTE, and the results are inconsistent. A case–control study found that fasting blood glucose levels did not increase the risk of VTE (OR = 0.98, 95% CI = 0.69–1.37) (25). However, another case–control study indicated that hyperglycemia is associated with an increased risk of VTE (OR = 2.21, 95% CI = 1.2–4.05) (26), which is consistent with our findings. Hyperglycemia activates coagulation through endothelial glycocalyx damage, upregulation of tissue factor, increased non-enzymatic glycosylation, and oxidative stress (27), thereby increasing the probability of VTE occurrence. Elevated triglyceride levels can promote VTE through multiple mechanisms. Firstly, hypertriglyceridemia can lead to increased blood viscosity, thereby slowing blood flow and increasing the risk of thrombus formation (28). Secondly, elevated triglyceride levels may further promote thrombus formation by affecting platelet activity and the expression of coagulation factors (29). Huang et al. (28) indicated that the combination of decreased high-density lipoprotein cholesterol (HDL-C) and elevated triglyceride levels significantly increases the risk of venous thromboembolism (VTE) formation. Given the close relationship between triglyceride levels and VTE formation, the management of hypertriglyceridemia should be strengthened in clinical practice (30).

In addition to the aforementioned factors, low albumin levels are an independent risk factor for VTE formation in severe ICH patients (31). Through Mendelian randomization analysis, the study further confirmed the relationship between low albumin levels and venous thrombosis (32). Albumin can modulate inflammatory responses, reducing the release of inflammatory factors, thereby lowering the risk of thrombus formation (33). Moreover, by binding to arachidonic acid, albumin inhibits its metabolism into potent platelet-aggregating substances such as thromboxane A2, thereby suppressing platelet activation and aggregation, and preventing thrombus formation (34). Albumin also improves vascular endothelial function by increasing the expression of nitric oxide (NO) and endothelial nitric oxide synthase (eNOS), thereby inhibiting thrombus formation (35). For patients with severe ICH who develop hypoalbuminemia, timely supplementation of albumin or implementation of other interventions may help reduce the risk of VTE occurrence (33, 35). Future research could further explore the specific mechanisms of albumin in thrombogenesis and its clinical application value.

Finally, the inflammatory response has also been confirmed to be involved in the formation of VTE. Studies have shown that elevated WBC levels are closely associated with an increased risk of VTE in patients with ICH (36), further corroborating our findings. The increase in WBC is typically related to systemic inflammatory responses, which can promote thrombosis by activating coagulation pathways and causing endothelial injury (37). In a study focusing on patients with neurological disorders, Makoto et al. explicitly identified WBC ≥ 7.6 × 10^9^/L as an independent predictor of DVT formation (38). In various diseases and clinical scenarios, the monitoring of WBC and its combined application with other biomarkers provide crucial evidence for the diagnosis, prediction, and prevention of venous thrombosis.

## 5 Conclusion

We have developed an interpretable XGBoost prediction model, which demonstrates exceptional performance in assessing the risk of VTE in critically ill ICH patients. Moreover, by quantifying the specific contributions of key pathophysiological indicators to the model’s predictions for individual patients through the SHAP framework, it enables personalized risk stratification and optimization of medical resource allocation.

## 6 Strengths

The strength of this study lies in the construction of a predictive model for VTE occurrence in severe ICH patients, addressing the limitation of traditional scoring methods that are not specifically tailored for ICH patients. In addition to conventional indicators such as length of hospital stay and age, the study also confirmed the independent predictive value of serum albumin, white blood cell count, and triglycerides for VTE occurrence in severe ICH patients. The model’s general applicability in the real world was supported by external validation from Qinghai Provincial People’s Hospital and decision curve analysis. Clinicians can utilize this model to identify high-risk patients and implement early individualized prevention. Furthermore, the application of the SHAP framework enhanced the model’s transparency, providing interpretable evidence for personalized interventions. The model can be integrated into clinical workflows (e.g., embedded in electronic medical record systems) to generate real-time risk scores, reducing reliance on subjective risk assessment tools such as the Padua score.

## 7 Limitations

This study has several limitations. Firstly, as a retrospective study based on publicly available data, its findings still require further validation through prospective studies. Secondly, the retrospective analysis itself may involve selection bias, which could affect the generalizability of the research results. Thirdly, the limited number of externally validated cases may affect the reliability of the study findings. Further validation in larger and more diverse cohorts is needed in the future. Fourthly, the MIMIC-IV database does not provide imaging features of intracerebral hemorrhage, such as hematoma volume, hematoma location, and intraventricular hemorrhage. In the future, if these imaging features of ICH become available, we will add relevant imaging information to this study and explore the value of these imaging features in patients with ICH combined with VTE.

## Data Availability

The original contributions presented in the study are included in the article/Supplementary material, further inquiries can be directed to the corresponding author.
