# Supplementary material for: Development of an interpretable machine learning model for predicting venous thromboembolism in intensive care unit patients with intracerebral hemorrhage
Source: Front Neurol. 2026 Jan 7;16:1691549. doi: 10.3389/fneur.2025.1691549 (PMC12819677; doi:10.3389/fneur.2025.1691549)
Supplement: Supplementary file 1 [file Table_1.docx]

| **Group** | **All data** | **Train data** | **Test data** | ***P*-value** |
| --- | --- | --- | --- | --- |
| **N** | 1545 | 1097 | 448 |  |
| **Gender** |  |  |  | 0.778 |
| Female | 692 (44.8%) | 198 (44.2%) | 494 (45.0%) |  |
| Male | 853 (55.2%) | 250 (55.8%) | 603 (55.0%) |  |
| **Atrial Fibrillation** |  |  |  | 0.666 |
| No | 1,095 (70.9%) | 314 (70.1%) | 781 (71.2%) |  |
| Yes | 450 (29.1%) | 134 (29.9%) | 316 (28.8%) |  |
| **CHF** |  |  |  | 0.822 |
| No | 1,286 (83.2%) | 375 (83.7%) | 911 (83.0%) |  |
| Yes | 259 (16.8%) | 73 (16.3%) | 186 (17.0%) |  |
| **Liver_Disease** |  |  |  | 0.623 |
| No | 1,408 (91.1%) | 411 (91.7%) | 997 (90.9%) |  |
| Yes | 137 (8.9%) | 37 (8.3%) | 100 (9.1%) |  |
| **Peripheral Vascular Disease** |  |  |  | 0.831 |
| No | 1,430 (92.6%) | 416 (92.9%) | 1,014 (92.4%) |  |
| Yes | 115 (7.4%) | 32 (7.1%) | 83 (7.6%) |  |
| **COPD** |  |  |  | 0.627 |
| No | 1,331 (86.1%) | 383 (85.5%) | 948 (86.4%) |  |
| Yes | 214 (13.9%) | 65 (14.5%) | 149 (13.6%) |  |
| **Paraplegia** |  |  |  | 0.954 |
| No | 940 (60.8%) | 272 (60.7%) | 668 (60.9%) |  |
| Yes | 605 (39.2%) | 176 (39.3%) | 429 (39.1%) |  |
| **Renal Disease** |  |  |  | 0.437 |
| No | 1,308 (84.7%) | 374 (83.5%) | 934 (85.1%) |  |
| Yes | 237 (15.3%) | 74 (16.5%) | 163 (14.9%) |  |
| **Malignant Cancer** |  |  |  | 0.926 |
| No | 1,391 (90.0%) | 403 (90.0%) | 988 (90.1%) |  |
| Yes | 154 (10.0%) | 45 (10.0%) | 109 (9.9%) |  |
| **VTE** |  |  |  | 0.877 |
| No | 1,305 (84.5%) | 380 (84.8%) | 925 (84.3%) |  |
| Yes | 240 (15.5%) | 68 (15.2%) | 172 (15.7%) |  |
| **Age** | 55.82 ± 17.99 | 55.67 ± 18.39 | 55.87 ± 17.84 | 0.926 |
| **Icu stay** | 6.42 ± 6.89 | 6.37 ± 6.69 | 6.44 ± 6.97 | 0.773 |
| **BMI** | 28.55 ± 7.39 | 28.45 ± 7.61 | 28.60 ± 7.29 | 0.849 |
| **Heart Rate** | 84.37 ± 18.32 | 83.66 ± 18.28 | 84.66 ± 18.34 | 0.467 |
| **SBP** | 133.47 ± 24.75 | 134.01 ± 24.65 | 133.26 ± 24.80 | 0.461 |
| **DBP** | 76.05 ± 19.20 | 75.02 ± 17.88 | 76.47 ± 19.70 | 0.235 |
| **Respiratory Rate** | 19.21 ± 5.24 | 19.20 ± 5.19 | 19.22 ± 5.26 | 0.897 |
| **Body Temperature** | 36.80 ± 0.77 | 36.74 ± 0.78 | 36.82 ± 0.77 | 0.429 |
| **Spo2** | 97.24 ± 3.41 | 97.31 ± 3.00 | 97.21 ± 3.56 | 0.889 |
| **BUN** | 21.54 ± 16.01 | 21.90 ± 17.33 | 21.39 ± 15.44 | 0.494 |
| **Serum Potassium** | 4.05 ± 0.67 | 4.08 ± 0.67 | 4.04 ± 0.68 | 0.334 |
| **Serum Sodium** | 139.09 ± 5.01 | 139.19 ± 5.05 | 139.05 ± 4.99 | 0.842 |
| **Blood Glucose** | 146.99 ± 64.16 | 147.19 ± 64.10 | 146.90 ± 64.21 | 0.772 |
| **Cr** | 1.19 ± 0.95 | 1.23 ± 1.03 | 1.17 ± 0.91 | 0.870 |
| **WBC** | 11.35 ± 5.70 | 11.69 ± 5.86 | 11.22 ± 5.63 | 0.139 |
| **RDW** | 14.42 ± 2.00 | 14.35 ± 2.05 | 14.44 ± 1.99 | 0.139 |
| **RBC** | 4.01 ± 0.79 | 4.05 ± 0.78 | 3.99 ± 0.79 | 0.115 |
| **Platelet Count** | 209.65 ± 91.18 | 209.45 ± 89.84 | 209.74 ± 91.76 | 0.960 |
| **MCV** | 91.11 ± 7.00 | 90.96 ± 6.38 | 91.17 ± 7.25 | 0.554 |
| **Hemoglobin** | 11.99 ± 2.29 | 12.13 ± 2.28 | 11.94 ± 2.30 | 0.125 |
| **Hematocrit** | 36.25 ± 6.52 | 36.64 ± 6.50 | 36.10 ± 6.52 | 0.089 |
| **MCH** | 30.08 ± 2.63 | 30.08 ± 2.45 | 30.09 ± 2.70 | 0.891 |
| **MCHC** | 33.04 ± 1.59 | 33.08 ± 1.56 | 33.03 ± 1.60 | 0.455 |
| **INR** | 1.34 ± 0.83 | 1.38 ± 1.20 | 1.32 ± 0.62 | 0.848 |
| **PT** | 14.27 ± 4.81 | 14.31 ± 4.95 | 14.25 ± 4.75 | 0.754 |
| **PTT** | 30.92 ± 10.36 | 31.34 ± 11.53 | 30.75 ± 9.84 | 0.773 |
| **Albumin** | 3.36 ± 0.70 | 3.33 ± 0.72 | 3.37 ± 0.69 | 0.310 |
| **Serum Calcium** | 8.63 ± 1.06 | 8.62 ± 1.12 | 8.63 ± 1.03 | 0.534 |
| **Triglycerides** | 162.57 ± 103.55 | 165.07 ± 105.41 | 161.55 ± 102.81 | 0.390 |
| **GCS** | 13.99 ± 2.08 | 14.05 ± 1.97 | 13.96 ± 2.12 | 0.520 |
| **SOFA** | 1.35 ± 1.93 | 1.39 ± 1.99 | 1.33 ± 1.90 | 0.448 |
